# The validation of culturally appropriate scales to assess the family health climate in a multi-ethnic Asian population

**DOI:** 10.3389/fpubh.2022.988525

**Published:** 2022-10-06

**Authors:** Yi-Ching Lynn Ho, Mary Su-Lynn Chew, Clement Zhong-Hao Ho, Aisyah Binte Latib, Vivian Shu-Yi Lee, Gladis Jing Lin, Julian Thumboo, Kinjal Doshi

**Affiliations:** ^1^Centre for Population Health Research and Implementation, Singapore Health Services, Singapore, Singapore; ^2^Program in Health Services and Systems Research, Duke-NUS Medical School, Singapore, Singapore; ^3^Department of Rheumatology and Immunology, Singapore General Hospital, Singapore, Singapore; ^4^Medicine Academic Clinical Program, Duke-NUS Medical School, Singapore, Singapore; ^5^Department of Neurology, Singapore General Hospital, Singapore, Singapore

**Keywords:** family health climate, instrument validation, physical activity, nutrition, health promotion, health behaviors, family

## Abstract

**Background:**

The Family Health Climate (FHC) is a family environment attribute postulated to influence the health behaviors of family members. It can be measured by domain scales for physical activity (FHC-PA) and nutrition (FHC-NU), which have been validated and used to identify health climate patterns in families in Western populations. To extend the use of the scales to Asian settings, this study aimed to adapt and validate the instruments for use in the multi-ethnic population of Singapore, accounting for language and cultural differences.

**Methods:**

In Part A (*n* = 40) to adapt the scales for the Singapore population, we performed cognitive interviews, face validity testing and pre-testing of the instruments (*n* = 40). Besides English, the scales were translated into Chinese and Malay. In Part B (*n* = 400), we performed exploratory and confirmatory factor analyses respectively on two random samples. We also tested for item discriminant validity, internal consistency reliability, construct validity, and measurement invariance.

**Results:**

The findings from the cognitive interviews in Part A led to scale adaptations to accommodate cultural and linguistic factors. In Part B, EFA on Sample I resulted in a three-factor model for the PA scale (accounting for 71.2% variance) and a four-factor model for the NU scale (accounting for 72.8% variance). CFA on Sample II indicated acceptable model fits: FHC-PA: χ^2^ = 192.29, *df* = 101, *p* < 0.001, χ^2^/*df* = 1.90; SRMR = 0.049; RMSEA = 0.067; CFI = 0.969; TLI = 0.963; FHC-NU: χ^2^ = 170.46, *df* = 98, *p* < 0.001, χ^2^/*df* = 1.74; SRMR = 0.036; RMSEA = 0.061; CFI = 0.967; TLI = 0.960. The scores of family members demonstrated significant agreement on the FHC-PA (Sg) [ICC_(2, 2)_ = 0.77] and FHC-NU (Sg) [ICC_(2, 2)_ = 0.75] scales. Findings suggest good evidence for item discriminant validity, internal consistency reliability, construct validity, and measurement invariance. Short versions of the scales were also developed.

**Conclusion:**

We adapted, translated and validated the scales for assessing the health climate of families in Singapore, including the development of short versions. The results showed good psychometric properties and the constructs had significant relationships with health behaviors and routines. Improving our understanding of family influences on individual health behavior will be important in developing multi-level strategies for health promotion and chronic disease prevention.

## Introduction

Lifestyle behavior interventions are necessary to address the increasing trend of non-communicable chronic diseases (1). Yet intervening in the individuals' lifestyles alone may not be the most effective, for one's personal choices and behavior are also influenced by interactions with environmental factors (2). The family is one important environmental determinant for health behaviors, with studies showing that behavior-related risk factors tend to aggregate within families (3) and findings of spousal concordance for chronic diseases that point to the influence of shared environments, beyond that of shared genetic risk (4–6).

In a shared environment like the family, there are collectively-held opinions, attitudes, feelings, and behaviors that are attributes of life in the social setting, and which may be termed the “climate” (7, 8). The climate may arise in the group of family members through their frequent interaction with each other and the reciprocal influencing of each other over an extended period of time. Based on this concept, i.e., the Model of Family Reciprocal Determinism (9, 10), the Family Health Climate (FHC) has been defined as the perceptions and cognitions concerning health and health behavior that are shared among family members (11). The FHC may be seen as a health behavior framework for an individual family member, through the individual's experience of daily family life, the discussion of health-related topics and family expectations concerning health values, behavior routines and interaction patterns within the family. Through the FHC, references are provided to members for valuing and interpreting their own behavior and that of others, therefore the FHC is an aspect of the family environment that shapes the daily health behaviors of the family members (12–14).

Niermann et al. (11) developed a set of questionnaires to assess a family's health climate in the domains of physical activity (PA) and nutrition (NU). These scales have been tested and validated in the German population. They have provided promising results, as the FHC-PA and FHC-NU scores have been found to be associated with individual variables like healthy eating, physical activity, food parenting practices and children's BMI (15, 16), suggesting relationships between family system influences on children and adolescent health behaviors. The findings also provided evidence for FHC as a family-level variable and can provide insight into how families influence each other's individual lifestyle behaviors. The recent identification of different clusters of families with specific co-occurring health behavioral patterns using the FHC scales (17) allows for targeted approaches to health promotion within the family. To date, the FHC scales have been applied largely in Europe and the USA and more recently validated for the Iranian and Turkish populations (18, 19). To apply the scales in Asian populations, there will be a need to adapt and validate the scales given sociocultural and language differences.

In this study, we aimed to adapt and validate the psychometric properties of the scales for the multi-ethnic population in Singapore. We hypothesized that there will be positive correlations of the respective FHC domains with family lifestyle behavior (frequency of family physical activities and family meals, encouragement for healthy lifestyles, and availability of healthy foods in the household) and to a lesser extent with individual lifestyle behavior (amount of physical activity and healthy food intake). We also hypothesized that there would a negative relationship between the FHC-NU scores and the household availability of unhealthy foods. Since the FHC scales had previously been shown to be a family level variable (11), we also hypothesized that at least moderate inter-rater agreement between family members on FHC scores.

## Methods

The study had two parts: Part A included the translation of the FHC scales into Simplified Chinese and Malay, the testing of face validity and the pre-testing of the translated scales. Part B of the study comprised the testing of the item, scale and construct validity of the FHC scales. Short versions of the scales were also developed. The study was approved by the institution's ethics review committee (CIRB Ref. 2020/2195).

### Part A

#### Participants

Forty individuals were recruited through an institutional research panel mailing list and an advertisement posted on the institutional website. The inclusion criteria were: age minimum of 15 years, Singapore residents, and fluency in English, Mandarin or Malay. Exclusion criteria were mental or cognitive disorders, as this may confound the responses for the validation process.

#### FHC scales and procedures

The first round of face validity testing was performed on a group of English-speaking participants (*n* = 10). The semi-structured, cognitive interviews used a probing technique (20) and lasted approximately 1 hour each. We used the original English version of the FHC scales (11). In the FHC-PA scale, there are 14 items within three factors (*value, cohesion*, and *information*): the five items under *value* assess the importance of being physically active for the whole family; the five items under *cohesion* assess joint physical activities and having fun together during these activities, while the four items under *information* assess the extent to which the family searches for and shares information related to sports and exercise (11). The FHC-NU consists of 17 items within four factors (*value, cohesion, communication*, and *consensus*): the four items under value assess the family's emphasis on nutrition in daily life; the five items under *cohesion* assess the importance of eating together with other family members; the five items under *communication* assess family support for balanced diets, while the three items under *consensus* assess the agreement of family members regarding daily eating behavior (11).

Based on feedback from the first round of testing, we modified and adapted the items for better understanding amongst the local population. Translations were also made from the English version into Chinese and Malay using professional services [Translation authorization was obtained from the corresponding author of the FHC scales (11)]. Independent translators performed forward and backward translations for each language and differences were reconciled. The Chinese and Malay FHC scales were then presented to new groups of Chinese- and Malay-speaking participants (*n* = 10 respectively) for face validity testing (see Supplementary File 1 for the Chinese and Malay scales).

Lastly, pre-testing interviews with a new group of English-speaking participants (*n* = 10) were conducted with the locally adapted scales [“FHC-PA (Sg) and FHC-NU (Sg)”]. They were asked to respond to the items on the 4-point Likert scale and elaborate on their understanding of the items and the sufficiency of the Likert scale. The four-point Likert scale rating was: 0 = “strongly disagree”, 1 = “somewhat disagree”, 2 = “somewhat agree”, 3 = “strongly agree”.

#### Data analysis

Face validity was analyzed based on participant qualitative feedback and interview transcripts. Specifically, we considered whether the vocabulary and phrasing of the items in the three languages could be understood linguistically and semantically. For each scale item, the study team discussed and made decisions together to modify the wording of the items if necessary.

After pre-testing the modified scales, we analyzed the qualitative feedback and interview transcripts to evaluate the adequacy of the 4-point response scale, as well as the cultural thought processes elicited by the items. Items were further modified, if necessary, based on consensus from among the study team.

### Part B

### Participants

Two hundred family dyads (i.e., 400 individuals) were recruited from the research panel of a survey vendor and from online advertisements placed on Facebook. The inclusion criteria were: age 15 years and above, Singapore residents, and living in the same household. Single-person households and tenants were excluded, as they do not share essential living arrangements, e.g., food preparation. A minimum sample size of 200 individuals, or at least 10 individuals per item for validation have been recommended in the literature (21–24). With a total of 33 items for the FHC (Sg) scales [16 items for Physical Activity (PA) and 17 items for Nutrition (NU)], our sample size met both criteria.

### Procedures

Participants filled in the Singapore Family Health Climate scales [FHC (Sg)] and other self-reported measures related to physical activity and diet (described below) to test construct validity. This was done *via* an online survey platform. Data on the demographic characteristics of participants (age, sex, race, education, occupation, marital status, type of housing and household income) were also collected. English versions of the questionnaires were used.

### Measures

#### Singapore family health climate scales

Following the results of Part A of the study, we used Version 1 of the Singapore scales (see Supplementary File 2). The FHC-PA (Sg) consisted of three factors (*value, cohesion*, and *information*) with a total of 16 items. The FHC-NU (Sg) consisted of four factors (*value, cohesion, communication*, and *consensus*), with a total of 17 items. The questionnaire used a four-point Likert scale rating of 0 = “strongly disagree”, 1 = “somewhat disagree”, 2 = “somewhat agree”, 3 = “strongly agree”. The range of possible scores for FHC-PA (Sg) and FHC-NU (Sg) were from 0 to 48 and 0 to 51 respectively, with higher scores indicating a better family health climate.

#### International physical activity questionnaire

We used the IPAQ, a 27-item questionnaire to assess the time spent by an individual on physical activity over the last seven days. The IPAQ has shown acceptable test-retest reliability and criterion validity across countries (25). We used the IPAQ scoring protocol for calculating physical activity-related energy expenditures (MET-minutes/week), focusing on the domain of recreation, sport, and leisure.

#### Diet screener

We used a 37-item diet screener (26) to assess the frequency of food eaten by an individual over the past year. It consists of items from the major food groups, such as grains, protein foods, dairy, fruits, vegetables, and foods high in sugar or fat, along with the types of drinks consumed. The diet screener has shown reasonable validity and good reproducibility when compared against the detailed Food Frequency Questionnaire in the Singapore population (26).

The Dietary Approaches to Stop Hypertension (DASH) scoring index (27) was used to calculate the intake of seven food groups: whole grains, fruits, vegetables, nuts and legumes, low fat dairy, red processed meats (reversed scoring), and sweet beverages (reversed scoring). Sodium was excluded from the total DASH score, as the diet screener may not accurately assess it, given the wide variation of sodium content in Asian dishes (26). DASH scores range from 7 to 35, with higher scores indicating higher consumption frequency of healthy food and/or lower consumption frequency from unhealthy food in comparison with other participants.

#### Family-related lifestyle behaviors

As part of construct validity testing, we probed family routines and support for healthy lifestyles and the availability of healthy foods (11). Specifically, we looked at the frequency of family engagement in physical activities or meals together, the frequency of encouragement by family members for partaking in physical activities or eating healthily, and the frequency of healthy and unhealthy foods made available in the household. A five-point Likert scale (1 = “Never” to 5 = “Very often”) was used.

### Data analysis

The dataset was divided into two samples to perform exploratory factor analysis (EFA) and confirmatory factor analysis (CFA), based on stratification of demographic characteristics: age [ < or ≥ median age of sample (41 years)], sex (male, female), and education (below tertiary level, tertiary level and above). Within these stratifications, random allocation to the two samples was done using a random number generator, resulting in *n* = 200 for each sample. There was no missing data, as the online survey platform would prompt for missing responses. STATA version 16.0 (STATA Corp LLC, TX, USA) was used for EFA and CFA. The rest of the data analysis was performed using IBM SPSS Statistics for Windows version 26.0 (IBM Corp., NY, USA).

The suitability of running an EFA was assessed through the Kaiser-Meyer-Olkin (KMO) measure of sampling adequacy, yielding an acceptable KMO statistic ≥ 0.70 (28) and a statistically significant χ^2^ value upon the Bartlett's Test of Sphericity (29). We then performed EFA on Sample I using principal axis factor extraction with oblique promax rotation (30, 31) to explore the underlying factorial structures in both FHC-PA and FHC-NU. The number of factors to be extracted was based on an initial eigenvalue threshold of 0.80 (31) and guided also by the structure of the original FHC factor model (PA: 3 factors, NU: 4 factors) (11). The criteria to retain items were: >0.40 for factor loadings, ≥0.4 corrected item-total correlations, and <0.4 communality (32). Skewness and kurtosis for the PA and NU scales were within the thresholds of 0 and 7 respectively (33). Visual inspections of the Q-Q plots for the FHC-PA and FHC-NU scores were done to assess normality and outliers. All 200 observations for FHC-PA were kept. One outlier (>3 *SD*) from the FHC-NU scores was removed.

To verify the factor structures obtained, CFA was performed on Sample II using independent cluster models. The commonly recommended fit indices χ^2^/*df*, TLI, CFI, SRMR and RMSEA were used to assess the overall goodness-of-fit. A good fit is indicated by χ^2^/*df* < 5, SRMR ≤ 0.08, RMSEA ≤ 0.06, CFI ≥ 0.95 and TLI ≥ 0.95 (34), while CFI ≥ 0.90, SRMR <0.10 and RMSEA <0.08 are considered adequate fits (34, 35).

We tested the assumptions of item scoring and the summated rating scales with the IQOLA Project Approach (36). The main assumptions tested were: (1) items in each factor contain a similar amount of information as the construct under examination; (2) items have homogeneous variances so that they contribute equally to the total score; (3) items are linearly related to the total score. Using both samples, we looked at the similarity of item means and standard deviations within each factor. We computed corrected item-total correlations after respectively removing items of interest from their respective factor scores to avoid inflated correlations. A threshold of 0.40 for the item-total correlations indicated item internal consistency. Item discriminant validity was tested by looking at whether item-total correlations were significantly higher for the corresponding factor than for competing factors. We also measured the internal consistency reliability of the factor items, i.e., how much the items in the factor co-vary relative to their sum score. Cronbach's alpha with a threshold of 0.70 was used.

To test construct validity, we assessed the correlations of the FHC scores with related measures on family routines, family support for healthy lifestyle, availability of healthy foods, using Samples I and II. Interpretation of Pearson's correlation coefficient (37) were as follows: *r* < 0.30, weak; 0.3 ≤ *r* < 0.49, moderate; 0.50 ≤ *r* < 1.00, strong correlation.

To ensure that the scales are measuring the same constructs across demographic groups, tests of measurement invariance (configural, metric and scalar) for age, sex, and education levels were performed on both samples. Configural invariance would be supported through finding equivalent numbers of factors and similar loadings of items onto their respective factors (38). Metric invariance would be analyzed by constraining factor loadings to be equivalent in the groups and comparing the model fit with that of the configural invariance model (i.e., unconstrained model) (38). The following thresholds determined metric invariance (Chen, 2007): a change of ≤ −0.010 in CFI, together with a RMSEA change of ≤ 0.015 or a SRMR change of ≤ 0.030. Upon support of metric invariance, we proceeded to test scalar invariance by constraining item intercepts to be equivalent among the groups (38). The thresholds for scalar invariance were: a change of ≤ −0.010 in CFI, together with a RMSEA change of ≤ 0.015 or a SRMR change of ≤ 0.010 (39). If non-invariance was found, we would investigate the source of non-invariance by unconstraining item loadings or intercepts and retesting the model to achieve a partial invariance model (38).

Finally, to assess the FHC (Sg) as a family-level variable, we used intraclass correlations to measure the concordance of responses among family dyads. A two-way, random-effects ICC model based on average measures between the dyads was used to assess the absolute agreement of FHC (Sg) scores for each dyad. Mean estimations along with 95% confidence intervals (CI) were reported for each ICC. Interpretations of ICC values were as follows: ICC < 0.50, poor; 0.50 ≤ ICC < 0.75, moderate; 0.75 ≤ ICC < 0.90, good; ICC > 0.90, excellent agreement (40).

After testing the full scales, short versions were developed (see Supplementary File 3 for details).

## Results

### Part A: Face validity and pre-testing

The participants' mean age was 40 years (*SD* = 12.5; range 20–71 years old). 75% were female, 72.5% were Chinese, 25% were Malay, 2.5% were of other races, and 80% had tertiary education. A variety of family household types were represented: 1-generation (12.8%), 2-generation (71.8%), 3-generation (15.4%), with an average of four persons living in one household.

The cognitive interviews elicited feedback on the wordings and understanding of the items, which resulted in the following changes: (1) Examples of physical activity and nutrition-related behavior were specified in the first item of each scale to orientate participants to the relevant contexts and to standardize thinking of what physical activity or nutrition might mean, especially in the local context. (2) Items that pertained to obtaining physical activity or nutrition information were updated to account for trends in searching for information online, e.g., “read newspaper or magazine articles” were modified to include both printed and online material. Similarly, “watch TV-programmes” was amended to “watch videos (e.g., on YouTube, Netflix, TV)” (3) Words and phrases were modified for better local understanding and interpretation of the scale items: The word “healthful” was changed to “healthy”. “Leisure time” was changed to “free time”. “It goes without saying” was simplified to “It is normal”. The phrase “like being together” was changed to “enjoy our time together”. The participants viewed physical activity and sports as distinct from each other; thus, we tested separate items with these words for uniqueness of contribution to our final scale. The original examples of sports provided were also changed from “bike tours and hikes” to “cycling, ball games, canoeing”, as highlighted by some participants that the original examples were not commonly played sports by families in Singapore. The appending of the word, “healthy” to items on nutrition and diet served to orientate participants to thinking of “healthy diet” and “healthy nutrition”, since there was feedback that the words “diet” and “nutrition” did not necessarily have positive meanings. Participants found it difficult to answer one of the original items under the *consensus* factor, “In our family, we rarely argue about food- or diet-related matters.” Some participants felt that the word “argue” was too strong a word to use and carried negative connotations, while others felt that some families may not argue to avoid conflict despite not agreeing.

Modifications to the wording of the original four-point Likert scale were made for easier understanding while retaining the graded meanings: The original version (0 = “definitely false”, 1 = “rather false”, 2 = “rather true”, 3 = “definitely true”) was modified to the following (0 = ”strongly disagree”, 1 = “somewhat disagree”, 2 = “somewhat agree”, 3 = “strongly agree”).

Two items were added to the FHC-PA (Sg) to reflect concepts that were measured in the original FHC-NU but not the original FHC-PA (“In our family, we encourage and support each other to be physically active”; “In our family, we usually agree on physical activities to do together”). At this stage, the FHC-PA (Sg) consisted of 16 items and the FHC-NU (Sg) consisted of 17 items. These “Version 1” scales can be found in Supplementary File 2. The score range for the FHC-PA (Sg) was 0 to 48, while the range for the FHC-NU (Sg) was 0 to 51.

### Part B: Factor structure and validity for item, scale, and constructs

The participants comprised 200 dyads from the same household and their relationships were: 43% parent-child, 37.5% couples, 19% siblings, and 0.5% aunt-nephew. 83% of the dyads lived in public housing and the remainder in private housing. 50.5% had household income between ≤ $7500. The mean age was 42 years (*SD* = 15.18; range 15–85 years old). 62.7% were female, 86.5% were Chinese, 6.0% were Malay, 6.3% were Indian, and 67.3% had tertiary education. There was a larger representation of female participants (62.7% vs. 51.1%) and those with tertiary education (67.3% vs. 32.4%), as compared to the population (41).

### Exploratory factor analysis

Sample I data for both sets of scale items were suitable for EFA as shown by the following statistics: FHC-PA (Sg): KMO statistic = 0.93; Bartlett's Sphericity Test χ^2^(120) = 2963.28, *p* < 0.001. FHC-NU (Sg): KMO statistic = 0.92; Bartlett's Sphericity Test χ^2^(136) = 2832.03, *p* < 0.001.

#### FHC-PA (Sg) scale

Three factors with eigenvalues ranging from 1.16 to 8.93 were identified and extracted, accounting for 71.7% of the variance. The three factors corresponded to the original factors of *cohesion, value* and *information*. The factor *cohesion*, which consists of items on family members engaging in physical activities together and the level of enjoyment of physical activity experienced together as a family, accounted for 55.8% of variance (6 items, eigenvalue = 8.93). The factor *value* contains items that represent the importance of physical activity in the family, and accounted for 8.6% of variance (6 items, eigenvalue = 1.38). Lastly, the factor *information*, which contains items on the use and collection of information materials relevant to physical activity in the family, accounted for 7.3% of variance (4 items, eigenvalue = 1.16). Factor loadings ranged from 0.54 to 0.97 (Table 1) and all items met the retention criteria. The two newly included items, which focused on encouragement and support to be physically active and agreement on doing physical activities together, loaded on the factors of *value* and *cohesion* respectively. A family that values the importance of physical activity would naturally encourage and support one another to engage in physical activity. Likewise, a family who is cohesive in terms of the climate on physical activity would likely agree more on exercising together.

**Table 1:** Item descriptives, factors, and item parameters of FHC-PA (Sg).

		**Sample I (*****n*** = **200)**		**Sample II (*****n*** = **200)**		**Samples I *and* II (*n* = 400)**
**Label**	**Item Description *(In our family…)***	***M*** **(*SD*)**	**a_EFA_**	***M*** **(*SD*)**	**a_CFA_**	***r*** **_corr_**
Val1	...we make it a point of being physically active during our daily life (e.g., taking walks, exercising, playing sports)	2.01 (0.74)	0.87	1.77 (0.74)	0.82	0.81
Val2	...it is normal to be physically active on a regular basis	1.98 (0.75)	0.93	1.78 (0.74)	0.88	0.86
Val3	...it is normal for us that we exercise on a regular basis	1.84 (0.83)	0.92	1.71 (0.81)	0.90	0.87
Val4	...it is normal to be physically active in our free time	1.88 (0.72)	0.54	1.72 (0.70)	0.80	0.73
Val5	...we agree that physical activities are part of our daily life	2.01 (0.71)	0.79	1.82 (0.74)	0.83	0.82
Val6	…we encourage and support each other to be physically active	2.09 (0.72)	0.57	1.93 (0.75)	0.73	0.70
Coh1	...we like spending time together doing physical activities	1.80 (0.78)	0.65	1.62 (0.79)	0.91	0.86
Coh2	...we enjoy exercising together	1.77 (0.81)	0.85	1.55 (0.83)	0.92	0.89
Coh3	...we have fun doing physical activities together	1.87 (0.79)	0.97	1.70 (0.80)	0.92	0.89
Coh4	...we find it very pleasant to be together doing physical activities	1.90 (0.75)	0.92	1.73 (0.81)	0.88	0.86
Coh5	...we like spending time together in sports (e.g., cycling, ball games, canoeing)	1.59 (0.89)	0.77	1.43 (0.87)	0.76	0.76
Coh6	...we usually agree on physical activities to do together	1.79 (0.78)	0.61	1.62 (0.83)	0.86	0.81
Inf1	...we watch videos (e.g., on YouTube, Netflix, or TV) on fitness, physical activities, or exercise	1.53 (0.91)	0.67	1.49 (0.87)	0.61	0.57
Inf2	...we actively look for the latest information on physical activity and exercise to stay up to date	1.41 (0.82)	0.81	1.20 (0.83)	0.88	0.80
Inf3	...we collect information (e.g., download/bookmark online information, cut out print articles) on fitness, physical activity, and exercise	1.32 (0.81)	0.85	1.17 (0.81)	0.89	0.80
Inf4	...we read articles (printed or online) on fitness, physical activity, and exercise	1.52 (0.80)	0.85	1.31 (0.88)	0.85	0.75

M, mean; SD, standard deviation; r_*corr*_, corrected item-total correlations; a_*EFA*_, factor loadings of exploratory factor analysis; a_*CFA*_, factor loadings of confirmatory factor analysis; Val, Value; Coh, Cohesion; Inf, Information. Full range of Likert scale scoring for each item was used (Min = 0; Max = 3).

#### FHC-NU (Sg) scale

An initial iteration identified two factors with eigenvalues 2.58 and 7.92. One item (“In our family, we rarely argue about food- or diet-related matters”) was dropped as it had a communality >0.4 and did not meet the item retention criteria. To match the original structure, we specified four factors. The retained items loaded into the four factors, with factor loadings ranging from 0.42 to 0.97 (Table 2), and corresponded well with the items under the original factors of *value, communication, cohesion* and *consensus*. 72.8% of total variance was accounted for by the four factors. The factor *value*, which contains items on the importance of nutrition, accounted for 49.5% of total variance (4 items, eigenvalue = 7.92). The factor *communication* covers items on family members actively communicating and encouraging each other about healthy diets, and accounted for 16.2% of total variance (5 items, eigenvalue = 2.59). The factor *cohesion* is concerned with the frequency and importance of having family meals together, and accounted for 4.3% of total variance (5 items, eigenvalue = 0.70). Finally the factor *consensus* relates to the agreement of family members on food-related matters, and accounted for 2.7% of total variance (2 items, eigenvalue = 0.43). Although the eigenvalues for the *cohesion* and *consensus* factor did not meet the 0.80 threshold, the two factors were kept for the following reasons: they were relatively distinct (the correlations between the factors were smaller than their reliability coefficients (Table 3), indicating unique variance measured by the factors (42), their internal consistency was high (0.93 and 0.83 for *cohesion* and *consensus* respectively; Table 3), and their inclusion allowed us to follow the original interpretation of the FHC model more closely.

**Table 2:** Item descriptives, factors, and item parameters of FHC-NU (Sg).

		**Sample I (*****n*** = 199*)		**Sample II (*****n*** = **200)**		Samples I *and* II (*n* = 399*)
**Label**	**Item Description *(In our family…)***	***M*** **(*SD*)**	**a_EFA_**	***M*** **(*SD*)**	**a_CFA_**	***r*** **_corr_**
Val1	...a healthy diet is important to us (e.g., type and amount of food, meal timings)	2.35 (0.66)	0.75	2.24 (0.64)	0.80	0.76
Val2	...we pay attention to eating healthily	2.24 (0.67)	0.85	2.18 (0.61)	0.89	0.84
Val3	...we eat healthily on a regular basis	2.10 (0.71)	0.78	2.07 (0.64)	0.82	0.80
Val4	...it is normal for us to choose healthy foods	2.13 (0.72)	0.70	2.10 (0.61)	0.84	0.81
Com1	...we are interested in articles (print or online) on healthy nutrition	1.86 (0.78)	0.42	1.71 (0.81)	0.59	0.58
Com2	...we remind each other to pay attention to a healthy diet	2.20 (0.69)	0.66	2.16 (0.65)	0.81	0.75
Com3	...we talk about which foods are healthy	2.13 (0.71)	0.92	2.09 (0.73)	0.84	0.82
Com4	...we encourage and support each other to refrain from eating/drinking unhealthy things	2.18 (0.69)	0.54	2.18 (0.69)	0.77	0.72
Com5	...we talk about how to eat healthily	2.14 (0.72)	0.82	2.12 (0.64)	0.87	0.80
Coh1	...we value spending time together during meals	2.37 (0.70)	0.79	2.32 (0.66)	0.81	0.81
Coh2	...everybody enjoys having meals together	2.42 (0.68)	0.85	2.30 (0.65)	0.86	0.85
Coh3	...eating together is a part of our daily family life	2.31 (0.73)	0.94	2.19 (0.72)	0.84	0.83
Coh4	...we enjoy meals most when we sit at the same table.	2.36 (0.72)	0.97	2.28 (0.67)	0.87	0.86
Coh5	...we try to eat together as often as possible	2.40 (0.67)	0.75	2.29 (0.65)	0.79	0.75
Con1	...we agree on diet and nutrition	2.11 (0.70)	0.77	2.01 (0.61)	0.82	0.70
Con2	...we usually agree on meals and food choices	2.20 (0.62)	0.81	2.11 (0.57)	0.75	0.70

M, mean; SD, standard deviation; r_corr_, corrected item-total correlations; a_EFA_, oblique rotation of factor loadings of exploratory factor analysis; a_CFA_, factor loadings of confirmatory factor analysis. *One outlier was excluded from Sample I. Val, Value; Com, Communication; Coh, Cohesion. Full range of Likert scale scoring for each item was used (Min = 0; Max = 3).

**Table 3:** Cronbach alpha coefficients and inter-factor Pearson's correlations of FHC (Sg) scales.

**FHC-PA (Sg) Factors**	**Value**	**Cohesion**	**Information**
Factor 1: Value	(0.93)		
Factor 2: Cohesion	0.73	(0.95)	
Factor 3: Information	0.59	0.61	(0.87)	
**FHC-NU (Sg) Factors**	**Value**	**Communication**	**Cohesion**	**Consensus**
Factor 1: Value	(0.92)			
Factor 2: Communication	0.76	(0.89)		
Factor 3: Cohesion	0.42	0.45	(0.93)	
Factor 4: Consensus	0.57	0.55	0.55	(0.83)

Cronbach's alpha coefficients are presented in brackets along the diagonals.

### Confirmatory factor analysis

**Figure 1:**
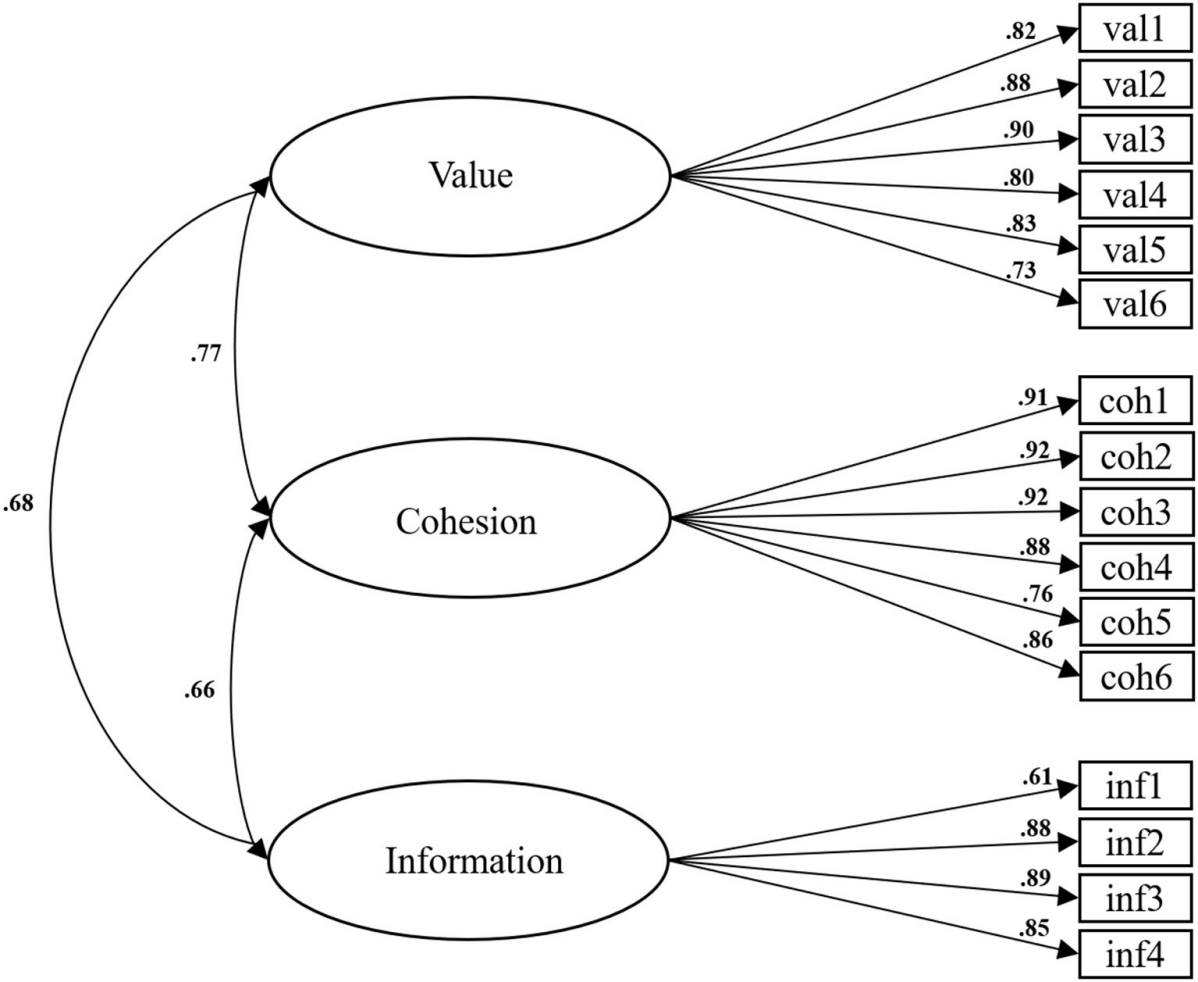
FHC-PA (Sg) standardized factor loadings and inter-factor correlations.

For FHC-PA (Sg), the fit indices (χ^2^ = 192.29, *df* = 101, *p* < 0.01, χ^2^/*df* = 1.90; SRMR = 0.049; RMSEA = 0.067; CFI = 0.969; TLI = 0.963) indicated an acceptable fit. Factor loadings, item-total correlations and inter-factor correlations are shown in Table 1 and Figure 1.

**Figure 2:**
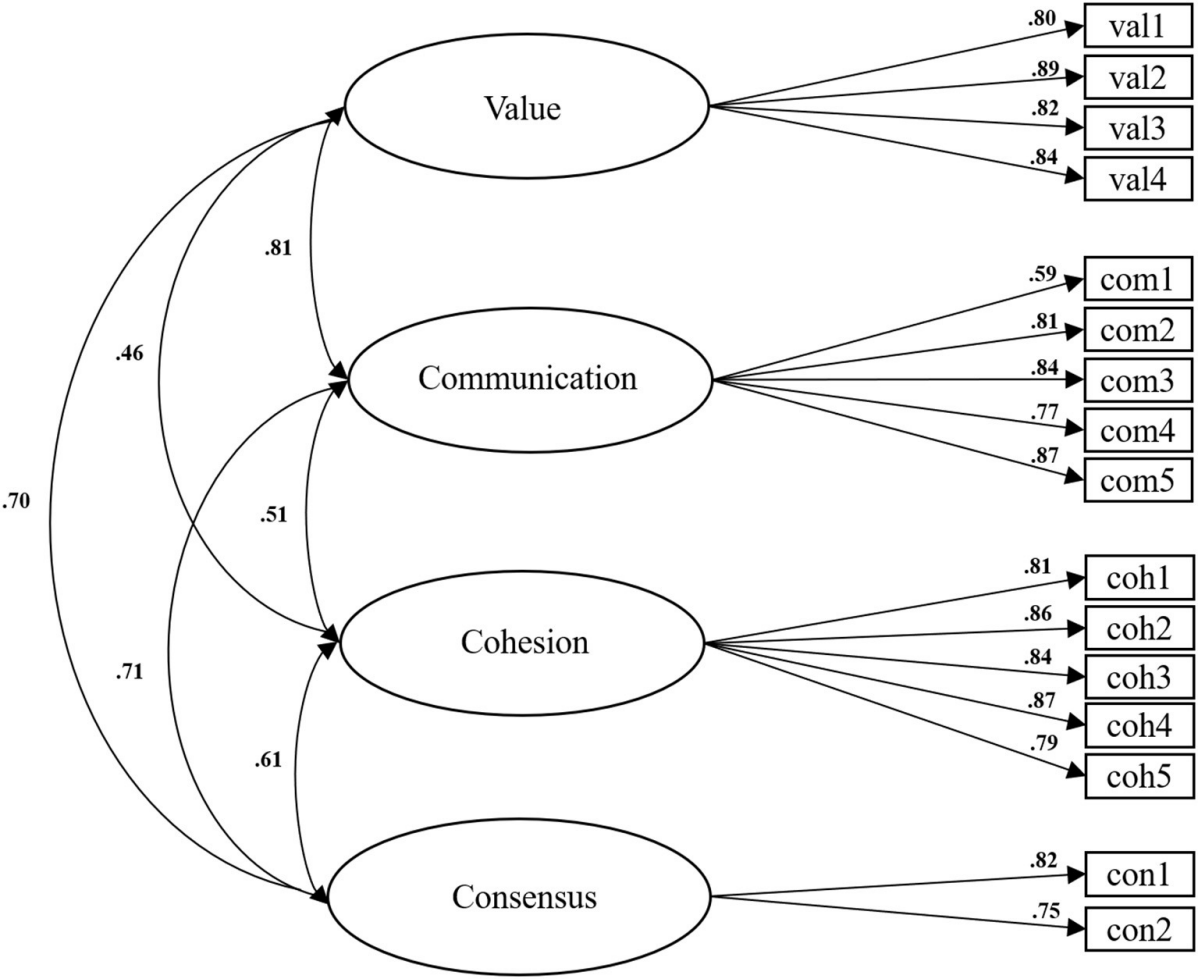
FHC-NU (Sg) standardized factor loadings and inter-factor correlations.

For FHC-NU (Sg), the fit indices (χ^2^ = 170.46, *df* = 98, *p* < 0.01, χ^2^/*df* = 1.74; SRMR = 0.036; RMSEA = 0.061; CFI = 0.967; TLI = 0.960) indicated an acceptable fit. Factor loadings, item-total correlations and inter-factor correlations are shown in Table 2 and Figure 2.

### Testing of assumptions for item scoring and the summated rating scales

The assumptions were supported. Item means and standard deviations were relatively similar within each factor (Tables 1, 2). The standard deviations for the items were below 1 and relatively homogeneous. The corrected item-total correlations for FHC-PA (Sg) and FHC-NU (Sg) were all greater than the threshold of 0.4 (Tables 1, 2). Item-total correlations of items were highest for their own scale (factor) as compared to correlations with other factors (Tables 4, 5). Item discriminant validity for both FHC-PA (Sg) and FHC-NU (Sg) were met (Tables 6, 7). Internal consistency of the factors met the Cronbach's alpha threshold of 0.7 (Table 3).

**Table 4:** Item-total correlations of FHC-PA (Sg) scale.

**FHC-PA (Sg)**		**Item-total correlations**		
**Label**	**Item description *(In our family…)***	**Factor 1 (Val)**	**Factor 2 (Coh)**	**Factor 3 (Inf)**
**Factor 1 = Value (Val**)				
Val1	...we make it a point of being physically active during our daily life (e.g., taking walks, exercising, playing sports)	0.81^*^	0.63	0.52
Val2	...it is normal to be physically active on a regular basis	0.86^*^	0.67	0.51
Val3	...it is normal for us that we exercise on a regular basis	0.87^*^	0.63	0.52
Val4	...it is normal to be physically active in our free time	0.73^*^	0.60	0.49
Val5	...we agree that physical activities are part of our daily life	0.82^*^	0.62	0.52
Val6	…we encourage and support each other to be physically active	0.70^*^	0.64	0.51
**Factor 2 = Cohesion (Coh)**				
Coh1	...we like spending time together doing physical activities	0.72	0.86^*^	0.58
Coh2	...we enjoy exercising together	0.68	0.89^*^	0.54
Coh3	...we have fun doing physical activities together	0.64	0.89^*^	0.50
Coh4	...we find it very pleasant to be together doing physical activities	0.66	0.86^*^	0.49
Coh5	...we like spending time together in sports (e.g., cycling, ball games, canoeing)	0.58	0.76^*^	0.53
Coh6	...we usually agree on physical activities to do together	0.66	0.81^*^	0.61
**Factor 3 = Information (Inf)**				
Inf1	...we watch videos (e.g., on YouTube, Netflix, or TV) on fitness, physical activities, or exercise	0.41	0.42	0.57^*^
Inf2	...we actively look for the latest information on physical activity and exercise to stay up to date	0.56	0.59	0.80^*^
Inf3	...we collect information (e.g., download/bookmark online information, cut out print articles) on fitness, physical activity, and exercise	0.51	0.55	0.80^*^
Inf4	...we read articles (printed or online) on fitness, physical activity, and exercise	0.54	0.51	0.75^*^

* Highest correlation among the factors. Item-total correlations have been corrected for overlap.

**Table 5:** Item-total correlations of FHC-NU (Sg) scale.

**FHC-NU (Sg)**		**Item-total correlations**			
**Label**	**Item description *(In our family…)***	**Factor 1 (Val)**	**Factor 2 (Com)**	**Factor 3 (Coh)**	**Factor 4 (Con)**
**Factor 1 = Value (Val)**				
Val1	...a healthy diet is important to us (e.g., type and amount of food, meal timings)	0.77^*^	0.63	0.38	0.49
Val2	...we pay attention to eating healthily	0.85^*^	0.70	0.37	0.52
Val3	...we eat healthily on a regular basis	0.80^*^	0.66	0.35	0.52
Val4	...it is normal for us to choose healthy foods	0.81^*^	0.73	0.38	0.51
**Factor 2 = Communication (Com)**				
Com1	...we are interested in articles (print or online) on healthy nutrition	0.52	0.57^*^	0.32	0.42
Com2	...we remind each other to pay attention to a healthy diet	0.67	0.75^*^	0.40	0.46
Com3	...we talk about which foods are healthy	0.65	0.82^*^	0.36	0.46
Com4	...we encourage and support each other to refrain from eating/drinking unhealthy things	0.65	0.72^*^	0.40	0.47
Com5	...we talk about how to eat healthily	0.70	0.81^*^	0.42	0.50
**Factor 3 = Cohesion (Coh)**				
Coh1	...we value spending time together during meals	0.35	0.39	0.81^*^	0.50
Coh2	...everybody enjoys having meals together	0.41	0.42	0.86^*^	0.54
Coh3	...eating together is a part of our daily family life	0.33	0.38	0.84^*^	0.47
Coh4	...we enjoy meals most when we sit at the same table	0.34	0.39	0.86^*^	0.47
Coh5	...we try to eat together as often as possible	0.43	0.43	0.76^*^	0.46
**Factor 4 = Consensus (Con)**				
Con1	...we agree on diet and nutrition	0.59	0.56	0.49	0.71^*^
Con2	...we usually agree on meals and food choices	0.46	0.46	0.53	0.71^*^

* Highest correlation among the factors. Item-total correlations have been corrected for overlap.

**Table 6:** Item-level discriminant validity testing for FHC-PA (Sg).

**FHC-PA (Sg)**					
**Label**	**Item Description (In our family…)**	**Mean**	**SD**	**Factor 1 (Val)**	**Factor 2 (Coh)**	**Factor 3 (Inf)**
**Factor = Val (Value)**					
Val1	...we make it a point of being physically active during our daily life (e.g., taking walks, exercising, playing sports)	1.89	0.75	–	2	2
Val2	...it is normal to be physically active on a regular basis	1.88	0.75	–	2	2
Val3	...it is normal for us that we exercise on a regular basis	1.78	0.82	–	2	2
Val4	...it is normal to be physically active in our free time	1.80	0.72	–	2	2
Val5	...we agree that physical activities are part of our daily life	1.91	0.73	–	2	2
Val6	…we encourage and support each other to be physically active	2.01	0.74	–	1	2
**Factor = Coh (Cohesion)**					
Coh1	...we like spending time together doing physical activities	1.71	0.79	2	–	2
Coh2	...we enjoy exercising together	1.66	0.83	2	–	2
Coh3	...we have fun doing physical activities together	1.78	0.80	2	–	2
Coh4	...we find it very pleasant to be together doing physical activities	1.82	0.78	2	–	2
Coh5	...we like spending time together in sports (e.g., cycling, ball games, canoeing)	1.51	0.88	2	–	2
Coh6	...we usually agree on physical activities to do together	1.70	0.81	2	–	2
**Factor = Inf (Information)**					
Inf1	...we watch videos (e.g., on YouTube, Netflix, or TV) on fitness, physical activities, or exercise	1.51	0.89	2	2	–
Inf2	...we actively look for the latest information on physical activity and exercise to stay up to date	1.31	0.83	2	2	–
Inf3	...we collect information (e.g., download/bookmark online information, cut out print articles) on fitness, physical activity, and exercise	1.24	0.81	2	2	–
Inf4	...we read articles (printed or online) on fitness, physical activity, and exercise	1.41	0.84	2	2	–

Cutoff point for significance is 2 standard errors (1 SE = 0.05, 2 SE = 0.1). Levels of scaling success: 2: Item-factor correlation is significantly higher for hypothesized scale than for competing scale. 1: Item-factor correlation is higher for hypothesized scale than competing scale, but not significantly.−1: Item-factor correlation is lower for hypothesized scale than competing scale, but not significantly.−2: Item-factor correlation is significantly lower for hypothesized scale than for competing scale.

**Table 7:** Item-level discriminant validity testing for FHC-NU (Sg).

**FHC-NU (Sg)**						
**Label**	**Item Description (In our family…)**	**Mean**	**SD**	**Factor 1 (Val)**	**Factor 2 (Com)**	**Factor 3 (Coh)**	**Factor 4 (Con)**
**Factor 1 = Val (Value)**						
Val1	...a healthy diet is important to us (e.g., type and amount of food, meal timings)	2.29	0.66	–	2	2	2
Val2	...we pay attention to eating healthily	2.20	0.65	–	2	2	2
Val3	...we eat healthily on a regular basis	2.08	0.68	–	2	2	2
Val4	...it is normal for us to choose healthy foods	2.11	0.67	–	1	2	2
**Factor 2 = Com (Communication)**						
Com1	...we are interested in articles (print or online) on healthy nutrition	1.79	0.80	1	–	2	2
Com2	...we remind each other to pay attention to a healthy diet	2.17	0.68	1	–	2	2
Com3	...we talk about which foods are healthy	2.10	0.73	2	–	2	2
Com4	...we encourage and support each other to refrain from eating/drinking unhealthy things	2.17	0.70	1	–	2	2
Com5	...we talk about how to eat healthily	2.12	0.69	2	–	2	2
**Factor 3 = Coh (Cohesion)**						
Coh1	...we value spending time together during meals	2.34	0.69	2	2	–	2
Coh2	...everybody enjoys having meals together	2.35	0.67	2	2	–	2
Coh3	...eating together is a part of our daily family life	2.24	0.74	2	2	–	2
Coh4	...we enjoy meals most when we sit at the same table	2.31	0.71	2	2	–	2
Coh5	...we try to eat together as often as possible	2.34	0.67	2	2	–	2
**Factor 4 = Con (Consensus)**						
Con1	...we agree on diet and nutrition.	2.06	0.66	2	2	2	–
Con2	...we usually agree on meals and food choices.	2.15	0.61	2	2	2	–

Cutoff point for significance is 2 standard errors (1 SE = 0.05, 2 SE = 0.1). Levels of scaling success: 2: Item-factor correlation is significantly higher for hypothesized scale than for competing scale. 1: Item-factor correlation is higher for hypothesized scale than competing scale, but not significantly. −1: Item-factor correlation is lower for hypothesized scale than competing scale, but not significantly. −2: Item-factor correlation is significantly lower for hypothesized scale than for competing scale.

### Construct validity

From Table 8, it can be seen that FHC-PA (Sg) was strongly correlated with the frequency of family engaging in physical activity together (*r* = 0.65, *p* < 0.001), as well as higher frequency of family encouraging each other to exercise (*r* = 0.57, *p* < 0.001), supporting convergent construct validity. Total FHC-PA (Sg) was weakly associated with an individual's amount of recreational physical activity as computed by the total Metabolic Equivalent of Task (MET) per week (*r* = 0.16, *p* < 0.001).

**Table 8:** Pearson's correlations for FHC-PA (Sg) with family- and individual-level physical activity behaviors.

	**Items**	**Total FHC-PA (Sg)**	**Value**	**Cohesion**	**Information**
Family physical activity behaviors	How often does your family engage in physical activities together?	0.65^**^	0.56^**^	0.67^**^	0.45^**^
	How often do family members encourage each other to engage in physical activities?	0.57^**^	0.56^**^	0.52^**^	0.38^**^
Individual physical activity behavior	Weekly amount of recreational physical activity (Total MET-minutes/week)	0.16^**^	0.16^**^	0.11^*^	0.15^**^

* p < 0.05;

** p < 0.001. Numbers are rounded to 2 decimal places.

FHC-NU (Sg) scores were significantly correlated with independent measurements concerning diet and nutrition on both the family and individual levels, supporting convergent construct validity for this scale (Table 9). FHC-NU (Sg) scores were moderately to strongly correlated with the frequency of family meals (*r* = 0.44, *p* < 0.001), the frequency of encouraging each other to eat healthily (*r* = 0.62, *p* < 0.001), and the availability of healthy foods at home (*r* = 0.45, *p* < 0.001). As hypothesized, there was a negative correlation with the availability of unhealthy foods at home (*r* = −0.25, *p* < 0.001). On the individual-level, higher total FHC-NU (Sg) scores were moderately correlated with higher DASH scores that indicate healthier diets (*r* = 0.31, *p* < 0.001).

**Table 9:** Pearson's correlations for FHC-NU (Sg) with family- and individual-level nutrition behaviors.

	**Items**	**Total FHC-NU (Sg)**	**Value**	**Communication**	**Cohesion**	**Consensus**
Family nutrition behaviors	How often does your family have meals together each week?	0.44^**^	0.22^**^	0.25^**^	0.60^**^	0.29^**^
	How often do family members encourage each other to eat healthily?	0.62^**^	0.53^**^	0.59^**^	0.43^**^	0.44^**^
	How often are healthy foods (e.g., fruits and vegetables) available in the household?	0.45^**^	0.47^**^	0.37^**^	0.30^**^	0.33^**^
	How often are unhealthy foods (e.g., soft drinks, fried snacks) available in the household?	−0.25^**^	−0.34^**^	−0.20^**^	−0.10^*^	−0.23^**^
Individual nutrition behavior	Diet quality (DASH score)	0.31^**^	0.37^**^	0.30^**^	0.14^**^	0.18^**^

* p < 0.05;

** p < 0.001. Numbers are rounded to 2 decimal places.

### Measurement invariance

The tests of measurement invariance and their goodness-of-fits across sex, age and education levels are shown in Table 10. The mean scores and SDs for each scale and factor by the demographic groups can be found in Tables 11, 12. For the FHC-PA (Sg) scale, configural invariance was supported across sex, age and education levels. The metric invariance model (constrained factor loadings) was not significantly different from the configural invariance model (fully unconstrained) for sex (Δχ^2^ = 21.7, Δ*df* = 16, *p* = 0.15) and education levels (Δχ^2^ = 19.4, Δ*df* = 16, *p* = 0.25). Across age groups, the constrained and unconstrained models differed significantly in goodness of fit (Δχ^2^ = 28.7, Δ*df* = 16, *p* = 0.03). Five items, which were all from the *value* factor, contributed to the fit differences. We tested for partial metric invariance by unconstraining the factor loadings of these items while the rest of the items remained constrained. Partial metric invariance was confirmed (Δχ^2^ = 4.64, Δ*df* = 11, *p* = 0.95). For scalar invariance (comparing models with constrained vs. unconstrained item intercepts), it was supported for all groups: sex (Δχ^2^ = 19.8, Δ*df* = 16, *p* = 0.23), age (Δχ^2^ = 17.5, Δ*df* = 16, *p* = 0.36), and education levels (Δχ^2^ = 16.1, Δ*df* = 16, *p* = 0.45).

**Table 10:** Fit-indices of the models for testing of measurement invariance.

	**χ^2^**	***df***	**χ^2^/*df***	**CFI**	**RMSEA**	**90% CI**	**SRMR**	**BIC**	**m∧**	**Δχ^2^**	**Δ*df***	***p***	**ΔCFI**	**Δ RMSEA**	**Δ SRMR**	**Decision**
**FHC-PA (Sg):**																
**Female (n = 251) and Male (n = 149)**																
M1	476^**^	202	2.36	0.954	0.082	0.073–0.092	0.049	10100	–	–	–	–	–	–	–	–
M2	498^**^	218	2.28	0.953	0.080	0.071–0.089	0.068	10000	M1	21.7	16	0.152	−0.001	−0.002	0.019	Accept
M3	518^**^	234	2.21	0.952	0.078	0.069–0.087	0.069	9970	M2	19.8	16	0.231	−0.001	−0.002	0.001	Accept
**< 41 years old (n = 194) and ≥41 years old (n = 206)**																
M1	485^**^	202	2.40	0.953	0.084	0.074–0.093	0.050	10100	–	–	–	–	–	–	-	-
M2	514^**^	218	2.36	0.950	0.082	0.073–0.092	0.114	10000	M1	28.7^*^	16	0.026	−0.003	−0.002	0.064	Reject
M2a	490^**^	213	2.30	0.954	0.081	0.071–0.090	0.066	10000	M1	4.64	11	0.947	0.001	−0.003	0.016	Accept
M3	531^**^	234	2.27	0.950	0.080	0.071–0.089	0.115	9920	M2	17.5	16	0.356	0.000	0.000	0.000	Accept
**Below tertiary (n = 131) and Tertiary (n = 269)**																
M1	514^**^	202	2.55	0.948	0.088	0.079–0.097	0.056	10100	–	–	–	–	–	–	-	-
M2	534^**^	218	2.45	0.947	0.085	0.076–0.094	0.094	10000	M1	19.4	16	0.250	−0.001	−0.003	0.038	Accept
M3	550^**^	234	2.35	0.947	0.082	0.073–0.091	0.094	9950	M2	16.1	16	0.450	0.000	−0.003	0.000	Accept
**FHC-NU (Sg):**																
**Female (n = 251) and Male (n = 149)**																
M1	395^**^	196	2.01	0.961	0.071	0.061–0.081	0.047	8981	–	–	–	–	–	–	-	-
M2	408^**^	212	1.92	0.962	0.068	0.058–0.078	0.068	8898	M1	12.6	16	0.703	0.001	−0.003	0.021	Accept
M3	424^**^	228	1.86	0.962	0.066	0.056–0.075	0.068	8818	M2	16.0	16	0.456	0.000	−0.002	0.000	Accept
**< 41 years old (n = 194) and ≥41 years old (n = 206)**																
M1	395^**^	196	2.02	0.962	0.071	0.061–0.081	0.045	8500	–	–	–	–	–	–	-	-
M2	406^**^	212	1.92	0.963	0.068	0.058–0.078	0.076	8846	M1	11.2	16	0.800	0.001	−0.003	0.031	Accept
M3	424^**^	228	1.86	0.962	0.066	0.056–0.075	0.076	8768	M2	18.2	16	0.314	−0.001	−0.002	0.000	Accept
**Pre-tertiary (n = 131) and Tertiary (n = 269)**																
M1	369^**^	196	1.88	0.966	0.066	0.056–0.077	0.046	8971	–	–	–	–	–	–	-	-
M2	379^**^	212	1.79	0.967	0.063	0.052–0.073	0.066	8885	M1	10.57	16	0.835	0.001	−0.003	0.020	Accept
M3	398^**^	228	1.74	0.967	0.061	0.051–0.071	0.066	8808	M2	18.45	16	0.298	0.000	−0.002	0.000	Accept

All estimates are rounded off to 3 significant figures.

* p < 0.05;

** p < 0.001. M1 = Configural invariance model (Unconstrained). M2 = Metric invariance model (Fully constrained factor loadings only). M2a = Partial metric invariance model (Partially constrained factor loadings only). M3 = Scalar invariance model (Fully constrained factor loadings and fully constrained item intercepts). m∧, Model comparison for difference between model fits.

**Table 11:** Demographic group means and standard deviations for FHC-PA (Sg) scale and factor levels.

	**FHC-PA (Sg)**	**Factor 1 (Val)**	**Factor 2 (Coh)**	**Factor 3 (Inf)**
**Sex**				
Female (*n* = 251)	26.52 (10.00)	11.10 (3.96)	9.89 (4.44)	5.53 (2.82)
Male (*n* = 149)	27.52 (9.40)	11.52 (3.77)	10.64 (4.21)	5.36 (2.96)
**Age**				
Young (*n* = 194)	26.56 (9.07)	10.92 (3.40)	10.20 (4.21)	5.43 (2.92)
Old (*n* = 206)	27.21 (10.42)	11.57 (4.29)	10.14 (4.52)	5.50 (2.83)
**Education**				
Tertiary (*n* = 269)	27.09 (9.41)	11.35 (3.74)	10.31 (4.19)	5.43 (2.87)
Pre-tertiary (*n* = 131)	26.48 (10.53)	11.07 (4.19)	9.88 (4.71)	5.53 (2.88)

Standard deviations are indicated in brackets.

**Table 12:** Demographic group means and standard deviations for FHC-NU (Sg) scale and factor levels.

	**FHC-NU (Sg)**	**Factor 1 (Val)**	**Factor 2 (Comm)**	**Factor 3 (Coh)**	**Factor 4 (Con)**
**Sex**				
Female (*n* = 251)	35.00 (7.93)	8.69 (2.31)	10.51 (2.91)	11.59 (3.23)	4.21 (1.20)
Male (*n* = 149)	34.50 (7.80)	8.64 (2.50)	10.10 (3.10)	11.56 (2.90)	4.19 (1.13)
**Age**				
Young (*n* = 194)	34.52 (7.32)	8.52 (2.27)	10.19 (2.88)	11.66 (3.01)	4.15 (1.12)
Old (*n* = 206)	35.09 (8.37)	8.82 (2.48)	10.51 (3.08)	11.51 (3.20)	4.25 (1.22)
**Education**				
Tertiary (*n* = 269)	35.06 (7.57)	8.80 (2.33)	10.42 (2.91)	11.65 (3.02)	4.18 (1.17)
Pre-tertiary (*n* = 131)	34.32 (8.48)	8.41 (2.47)	10.22 (3.13)	11.44 (3.28)	4.25 (1.18)

Standard deviations are indicated in brackets.

For the FHC-NU (Sg) scale, configural invariance was supported across sex, age and education levels. For metric invariance, the constrained and unconstrained models did not differ significantly for sex (Δχ^2^ = 12.6, Δ*df* = 16, *p* = 0.70), age (Δχ^2^ = 11.2, Δ*df* = 16, *p* = 0.80) and education levels (Δχ^2^ = 10.6, Δ*df* = 16, *p* = 0.84), supporting metric invariance. Scalar invariance was also supported for sex (Δχ^2^ = 16.0, Δ*df* = 16, *p* = 0.46), age (Δχ^2^ = 18.2, Δ*df* = 16, *p* = 0.31) and education levels (Δχ^2^ = 18.45, Δ*df* = 16, *p* = 0.30).

### Intraclass correlations for family dyad scores

A good level of agreement was found between members of family dyads for FHC-PA (Sg) and there was a moderate level of agreement for FHC-NU (Sg). The average measure ICC_(2, 2)_ for FHC-PA (Sg) was 0.77, with a 95% CI from 0.70 to 0.83 [*F*_(199, 199)_ = 4.41, *p* < 0.001]. For FHC-NU (Sg), the average measure ICC_(2, 2)_ was 0.75 with a 95% CI from 0.67 to 0.81 [*F*_(199, 199)_ = 4.00, *p* < 0.001]. Moderate to good levels of agreement were also found comparing dyad scores for the individual factors of FHC-PA (Sg) and FHC-NU (Sg) (Table 13).

**Table 13:** Intraclass correlations (ICC) of FHC-Sg scales within family dyads.

	**ICC_(2, 2)_**	**(Lower Bound–Upper Bound)**
**FHC-Sg (PA)**		
FHC-Sg (PA) Scale (16 items)	0.77	(0.70–0.83)
FHC-Sg (PA) Factor 1 (6 items)	0.78	(0.71–0.83)
FHC-Sg (PA) Factor 2 (6 items)	0.75	(0.67–0.81)
FHC-Sg (PA) Factor 3 (4 items)	0.67	(0.56–0.75)
**FHC-Sg (NU)**		
FHC-Sg (NU) Scale (16 items)	0.75	(0.67–0.81)
FHC-Sg (NU) Factor 1 (4 items)	0.74	(0.66–0.80)
FHC-Sg (NU) Factor 2 (5 items)	0.71	(0.61–0.78)
FHC-Sg (NU) Factor 3 (5 items)	0.75	(0.67–0.81)
FHC-Sg (NU) Factor 4 (2 items)	0.67	(0.56–0.75)

All the ICC values ranged from moderate to good level of agreement (40). Samples I and II were used (n = 400).

## Discussion

Based on the original Family Health Climate scales developed by Niermann et al. (11), we have developed a culturally appropriate set of scales, including Chinese and Malay translations, for the multi-ethnic population in Singapore. We have tested the scales' psychometric properties and validated them from item to factor level and across demographic groups. The FHC (Sg) scores also demonstrated good inter-rater reliability within family dyads, supporting FHC (Sg) as a family-level variable.

### Language and cultural considerations

Given the multi-ethnic population in Singapore, the FHC scales were translated into Simplified Chinese and Malay, as this would cover up to 96.1% of the population who spoke English, Chinese, Chinese dialect or Malay at home (43) [Tamil, the fourth national language is spoken by 2.5% at home, the majority of whom are also able to communicate in English, thus we did not translate the FHC scales into Tamil (43)] Since the original FHC scales were translated from German (11), face validity and pre-testing interviews of the FHC (Sg) scales were conducted to examine potential differences in understanding and interpretations, and account for cultural influences on language encoding or decoding processes. An example is the perceived negative connotation attached to the word “argue”, in one of the items under the *consensus* factor, “In our family, we rarely argue about food- or diet-related matters”. Participants felt the word “argue” was too negative and that including it would not serve the purpose of the probe, since some Asian families, despite disagreeing, may not argue simply to avoid conflict. Asian cultures are known to be collectivistic and conflict avoidance is an associated characteristic, as compared to individualism in Western cultures (44). In line with the qualitative feedback received, we also found that this item had high communality and thus we eventually removed the item from the FHC-NU (Sg) scale.

### Factor structure of the FHC (Sg) scales

Overall the factor structures and item loadings of the FHC (Sg) scales show that the intended concepts of the original FHC scales did not differ much for the Singapore population. All three original factors (*value, cohesion, information*) for the FHC-PA were replicated in the Singapore version even with the inclusion of two new items (Val6 and Coh6), which were mirrors of FHC-NU items. The original four factors for the FHC-NU scale were also replicated (*value, communication, cohesion* and *consensus*) (11). The fourth factor, *consensus* currently has two items, as the third item on “…we rarely argue about food- or diet-related matters” was dropped, since it did not meet the item inclusion criteria and also received poor feedback during the cognitive interviews, as discussed in the section above. With two items remaining in the *consensus* factor, the removal of the factor altogether may be considered (45). We chose to retain the factor, because firstly, the concept of consensus is important when considering the nutrition “climate” of a family. Since a “climate” encompasses opinions, attitudes, feelings, and behaviors that are shared within a social group (7, 8), the factor of *consensus* (the agreement of family members on daily eating behavior) is an important contributor to the climate concept. Second, the goodness-of-fits for this model met acceptable thresholds and surpassed the fits for models with the same items forced into three factors, or models that did not drop the item (The results of these EFAs are not reported here, but can be requested from the authors). Third, the two items on the *consensus* factor are highly correlated with each other (*r* = 0.71) and less correlated with other items (*r* from 0.46 to 0.59; Tables 4, 5) (32). Future studies should nonetheless consider generating additional items for the *consensus* factor to strengthen its construct.

### Validity and reliability

Across various tests, the scales showed validity and reliability: The assumptions for item scoring and the summated rating scales were tested to be valid. There was internal consistency reliability for each factor, as shown by the high Cronbach alphas. Construct validity was supported: the FHC-PA (Sg) and FHC-NU (Sg) scores were strongly and positively correlated with independent measurements for family-level behavior concerning physical activity and nutrition (i.e., family routines for exercise and meals, the frequency of encouragement among family members for these behaviors, and the availability of healthy foods in the household). Conversely, the availability of unhealthy foods in the household was negatively correlated with the FHC-NU (Sg) score. These results show the close relationship of the FHC to health-related behavior of the family as a whole and provide support for the FHC as a family-level construct (11). The FHC is also expected to be related, albeit less strongly, to individual health behaviors; this is because even though the health climate of a family should influence one's health behavior, it is a broad, collective attribute that is distinct from objective, specific measurements of physical activity and nutrition for the individual (11, 46). Indeed we found that though the correlations were significant, it was relatively weak for the FHC-PA (Sg) with individual physical activity levels, while the FHC-NU (Sg) was moderately correlated with individual diet quality. It would be important for intervention strategies to understand the mechanisms by which the FHC may percolate within the family to influence individual members' health behaviors.

### Measurement invariance

Psychometric equivalence of the FHC-NU construct was found across age, sex and education levels, as demonstrated through configural, metric and scalar measurement invariance. For FHC-PA, there was similarly measurement invariance across the demographic groups, with the exception of partial metric invariance for age groups. The non-invariant items belonged to the value factor, meaning that the items do not contribute to the value construct in a similar degree across age. Caution may be needed when administering the FHC-PA (Sg) to a wide age range, even though the effects on the mean differences of the value factor are unlikely to be significant, given that simulations have shown that partial metric invariance generally has minimal effects (47).

### Relationship of dyadic scores

The ratings between family dyad members for FHC-PA (Sg) and FHC-NU (Sg) had at least moderate to good levels of agreement within pairings ranging from parent-child, couples, siblings and aunt-nephew, as indicated by the intraclass correlations [ICC_(2, 2)_]. Taken together with our findings of strong correlations between the scale scores with relevant family-level behaviors, it adds support to the concept of FHC as a family-level variable (11, 16). These results also provide confidence that a single family member's FHC rating is likely to be representative of the other family members (11, 48, 49).

As an aspect of family life, it would be necessary to find out how a healthy FHC is developed and how it in turn influences individuals. Work on this has started, e.g., Wasche et al., 2021 (50) have explored the underlying processes and mechanisms which create the FHC that influences family members' health behaviors. Further work in different cultural settings is needed, where family systems are likely to be different (51). Understanding the role of the family in health promotion and the models and mechanisms involved in families with varied characteristics will allow us to better develop family-based interventions to improve the health climate, individuals' lifestyles and their health (52).

### Strengths and limitations of study

As far as we are aware, this is the first study on the family health climate in Singapore and Southeast Asia, a region with a unique mix of cultures and languages, which necessitates the adaptation and translation of the FHC scales. As the Malay and Chinese languages are also used in several other population groups around Southeast Asia, the adapted and translated scales may prove useful for studies on family health climates in the region. Furthermore, with studies highlighting the differences in health and lifestyle behaviors among the various ethnicities in Singapore (53), the FHC instrument can help identify key, actionable constructs of family climates that may be common to target in promotion of healthier lifestyles.

The limitations in the study include the following: The generalisability of the FHC (Sg) scales could be limited by the over-representation of female participants and those with tertiary education, as compared to the population (41). However, this was mitigated by the tests of measurement invariance, which indicated the equivalence of the constructs across sex and education levels. Measurement invariance was not tested across ethnicity, because the group sizes were statistically unbalanced (86% Chinese ethnicity, with Malays (6%), Indians (6%) and other races forming the remainder in the Part B sample), and this would affect the accuracy of the results (54). The length of time the sample of family dyads lived together could be a factor in the FHC scores, but as there was no data on this, we cannot yet assess the role of this factor. Future studies could also be done to confirm the factor structures, internal reliability and construct validity of the face-valid Chinese and Malay versions, which were not used in Part B. Finally, the study data was collected during the COVID-19 period, which could have disrupted to some extent family and/or individual lifestyle behaviors and routines. While it is unlikely that such changes will significantly impact the FHC scores, since the family health climate is likely to be developed over time through family processes (11, 16, 49), the correlations of FHC scores with family- and individual-level health behaviors and routines could have been underestimated due to the pandemic's disruptions to daily life.

## Conclusion

This study has validated the use of the FHC (Sg) scales in English, together with Chinese and Malay translations, to enable assessments of the health climate of families in Singapore. Overall the results showed good psychometric properties, with the FHC constructs further shown to be family-level variables with strong relationships to family health behaviors and routines, and associated also with individual health behavior to a lesser degree. Short versions of the FHC (Sg) scales have also been developed for ease of use in future studies. Understanding the family influence on individual health behavior will be key in developing strategies for healthy lifestyle promotion and disease prevention.

## Data availability statement

The datasets presented in this article are not readily available because of the stipulations from the local institutional review committee on data sharing beyond the approved study team members. Requests to access the datasets should be directed to: lynn.ho@duke-nus.edu.sg.

## Ethics statement

The studies involving human participants were reviewed and approved by the Centralized Institutional Review Board (CIRB), Singapore Health Services. All subjects gave written informed consent, except in Part B of the study, where written informed consent was not required, as individuals had full choice to participate or leave the online survey at any point, and no personal identifiers were retained.

## Author contributions

Y-CH conceptualized and designed the study. KD guided the study. Y-CH and JT acquired funding for the study. MC, CH, AL, KD, and Y-CH conducted the multi-language cognitive interviews. MC did project administration, data charting, and visualization. MC, Y-CH, and VL performed the data analysis. GL guided the analysis of the diet screener. Y-CH and MC wrote the first draft. All authors contributed to manuscript revision, read, and approved the submitted version.

## Funding

This study was funded by a grant (CGDec19S11) from the SingHealth Regional Health System (PULSES) Center Grant, Singapore. Y-CH, MC, AL, VL, and GL were funded by a grant (NMRC/CG/C027/2017) from the National Medical Research Council, Singapore.

## Conflict of interest

The authors declare that the research was conducted in the absence of any commercial or financial relationships that could be construed as a potential conflict of interest.

## Publisher's note

All claims expressed in this article are solely those of the authors and do not necessarily represent those of their affiliated organizations, or those of the publisher, the editors and the reviewers. Any product that may be evaluated in this article, or claim that may be made by its manufacturer, is not guaranteed or endorsed by the publisher.

## Supplementary material

Supplementary File 1 FHC-PA and NU (Sg) Chinese and Malay Final Versions.

Supplementary File 2 FHC-PA and NU (Sg) Version 1.

Supplementary File 3 Development of FHC-PA and NU (Sg) Short Version.

## References

[1] Naghavi M Abajobir AA Abbafati C Abbas KM Abd-Allah F Abera SF. Global, regional, and national age-sex specific mortality for 264 causes of death, 1980–2016: a systematic analysis for the global burden of disease study 2016. Lancet. (2017) 390:1151–210. 10.1016/S0140-6736(17)32152-9 28919116 PMC5605883

[2] Bandura A. Social Foundations of Thought and Action: A Social Cognitive Theory. Englewood Cliffs, NJ: Prentice-Hall (1986).

[3] Campbell TL. Familien und Gesundheit: Zum Stand der Forschung [Families and health: The current state of research]. In: Kroger F, Hendrischke A, McDaniel SH, editors. Familie, System und Gesundheit Systemische Konzepte fur ein soziales Gesundheitswesen [Family, System and Health Systemic Concepts for a Social Health Care System]. Heidelberg: Auer (2000), p. 225–41.

[4] Agerbo E. Risk of suicide and spouse's psychiatric illness or suicide: nested case-control study. BMJ. (2003) 327:1025–6. 10.1136/bmj.327.7422.1025 14593038 PMC261658

[5] Hippisley-Cox J. Married couples' risk of same disease: cross sectional study. BMJ. (2002) 325:636. 10.1136/bmj.325.7365.636 12242177 PMC126307

[6] Sackett D Holland W. Controversy in the detection of disease. Lancet. (1975) 306:357–9. 10.1016/S0140-6736(75)92790-7 51154

[7] Von Rosenstiel L Betriebsklima und Leistung – Eine Wissenschaftliche Standortbestimmung [Working atmosphere and performance – A scientific assessment]. In: Haugenbrauck U., Kock K., Kutzner E., Muesmann G., editors. Handbuch Betriebsklima [Working Environment Manual]. Munchen: Rainer Hampp Verlag (2003), p. 23–8.

[8] Ekvall G. Organizational climate for creativity and innovation. Eur J Work Organ. (1996) 5:105–23. 10.1080/13594329608414845

[9] Baranowski T. Families and health actions. In: Gochman DS, editor. Handbook of Health Behavior Research 1: Personal and Social Determinants. New York: Plenum Press (1997), p. 197–206.

[10] Taylor W Baranowski T Sallis J. Family determinants of childhood physical activity: a social cognitive model. In: Dishman RK, editor. Advances in Exercise Adherence. Champaign, IL: Human Kinetics (1994), p. 319–42.

[11] Niermann C Krapf F Renner B Reiner M Woll A. Family health climate scale (FHC-Scale): development and validation. Int J Behav Nutr. (2014) 11:1–14. 10.1186/1479-5868-11-30 24593840 PMC4015295

[12] Brown T Smith S Bhopal R Kasim A Summerbell C. Diet and physical activity interventions to prevent or treat obesity in South Asian children and adults: A Systematic review and meta-analysis. Int J Environ. (2015) 12:566–94. 10.3390/ijerph120100566 25584423 PMC4306880

[13] Van Sluijs EMF Kriemler S McMinn AM. The effect of community and family interventions on young people's physical activity levels: a review of reviews and updated systematic review. Br J Sports Med. (2011) 45:914–22. 10.1136/bjsports-2011-090187 21836175 PMC3736309

[14] Bronfenbrenner U. Ecological Systems Theory. Six theories of Child Development: Revised Formulations and Current Issues. London: Jessica Kingsley Publishers (1992), p. 187–249.

[15] Gerards SMPL Niermann CYN Gevers DWM Eussen N Kremers SPJ. Context matters! The relationship between mother-reported family nutrition climate, general parenting, food parenting practices and children's BMI. BMC Public Health. (2016) 16:1–10. 10.1186/s12889-016-3683-8 27677380 PMC5039910

[16] Niermann CYN Kremers SPJ Renner B Woll A. Family health climate and adolescents' physical activity and healthy eating: a cross-sectional study with mother-father-adolescent triads. PLoS One. (2015) 10:1–18. 10.1371/journal.pone.0143599 26606157 PMC4659539

[17] Niermann CYN Spengler S Gubbels JS. Physical activity, screen time, and dietary intake in families: a cluster-analysis with mother-father-child triads. Front Public Health. (2018) 6:276. 10.3389/fpubh.2018.00276 30324100 PMC6172305

[18] Güney E Karataş Okyay E Uçar T. Families' health behavior: validity and reliability of the Turkish Version of the family health climate scale. Soc Work Public Health. (2021) 36:707–22. 10.1080/19371918.2021.1948484 34340641

[19] Kharazmi A Brant JM Sajjadi M Moshki M Sadegh Moghadam L. Validation of the persian version of family health climate scale (FHC-Scale) in Iranian families. BMC Public Health. (2020) 20:1–9. 10.1186/s12889-020-09931-8 33272246 PMC7713167

[20] Miller K Chepp V Willson S Padilla JL. Cognitive Interviewing Methodology. New York, NY: John Wiley and Sons (2014). 10.1002/9781118838860

[21] Boateng GO Neilands TB Frongillo EA Melgar-Quiñonez HR Young SL. Best practices for developing and validating scales for health, social, and behavioral research: a primer. Front Public Health. (2018) 6:149. 10.3389/fpubh.2018.00149 29942800 PMC6004510

[22] Comrey AL. Factor-analytic methods of scale development in personality and clinical psychology. J Consult Clin Psychol. (1988) 56:754–76. 10.1037/0022-006X.56.5.754 3057010

[23] Guadagnoli E Velicer WF. Relation of sample size to the stability of component patterns. Psychol Bull. (1988) 103:265–75. 10.1037/0033-2909.103.2.265 3363047

[24] Nunnally JC Bernstein IH. Psychometric Theory. 3rd Edn. New York, NY: McGraw-Hill (1994).

[25] Craig CL Marshall AL Sjöström M Bauman AE Booth ML Ainsworth BE. International physical activity questionnaire: 12-Country reliability and validity. Med Sci Sports Exerc. (2003) 35:1381–95. 10.1249/01.MSS.0000078924.61453.FB 12900694

[26] Whitton C Ho JCY Rebello SA van Dam RM. Relative validity and reproducibility of dietary quality scores from a short diet screener in a multi-ethnic Asian population. Public Health Nutr. (2018) 21:2735–43. 10.1017/S1368980018001830 30081973 PMC10260988

[27] Fung TT Hu FB Wu K Chiuve SE Fuchs CS Giovannucci E. The mediterranean and dietary approaches to stop hypertension (DASH) diets and colorectal cancer. Am J Clin Nutr. (2010) 92:1429–35. 10.3945/ajcn.2010.29242 21097651 PMC2980967

[28] Kaiser HF. An index of factorial simplicity. Psychometrika. (1974) 39:31–6. 10.1007/BF02291575

[29] Bartlett MSA. note on the multiplying factors for various chi-square approximations. J R Stat Soc Series B Stat Methodol. (1954) 16:296–8. 10.1111/j.2517-6161.1954.tb00174.x

[30] Briggs NE MacCallum RC. Recovery of weak common factors by maximum likelihood and ordinary least squares estimation. Multivariate Behav Res. (2003) 38:25–56. 10.1207/S15327906MBR3801_2 26771123

[31] Stark R. Guide to Decision-Making in Exploratory Factor Analysis. (2019). Available online at: https://www.researchgate.net/publication/334223802_Guide_to_Decision-Making_in_Exploratory_Factor_Analysis (accessed September 05, 2021).

[32] Worthington RL Whittaker TA. Scale development research. Couns Psychol. (2006) 34:806–38. 10.1177/0011000006288127

[33] Curran PJ West SG Finch JF. The robustness of test statistics to non-normality and specification error in confirmatory factor analysis. Psychol Methods. (1996) 1:16–29. 10.1037/1082-989X.1.1.16

[34] Hoyle RH. Handbook of Structural Equation Modeling. New York, NY: Guilford Press (2012).

[35] Bentler PM. Comparative fit indexes in structural models. Psychol Bull. (1990) 107:238–46. 10.1037/0033-2909.107.2.238 2320703

[36] Ware JE Gandek B. Methods for testing data quality, scaling assumptions, and reliability. J Clin Epidemiol. (1998) 51:945–52. 10.1016/S0895-4356(98)00085-7 9817111

[37] Cohen J. Statistical Power Analysis for the Behavioral Sciences. 2nd Edn. London: Routledge. (1988).

[38] Putnick DL Bornstein MH. Measurement invariance conventions and reporting: the state of the art and future directions for psychological research. Dev Rev. (2016) 41:71–90. 10.1016/j.dr.2016.06.004 27942093 PMC5145197

[39] Chen FF. Sensitivity of Goodness of Fit Indexes to Lack of Measurement Invariance. Structural Equation Modeling. Newark, DE: Taylor and Francis. (2007).

[40] Koo TK Li MYA. Guideline of selecting and reporting intraclass correlation coefficients for reliability research. J Chiropr Med. (2016) 15:155–63. 10.1016/j.jcm.2016.02.012 27330520 PMC4913118

[41] Singapore Department of Statistics. Population trends (2020). Available online at: https://www.singstat.gov.sg/publications/population/population-trends (accessed September 27, 2021).

[42] Guilford JP. Psychometric Methods. 2nd Edn. New York: McGraw-Hill (1954).

[43] Singapore Department of Statistics. Singapore Census of Population 2020, Statistical Release 1: Demographic characteristics, education, language and religion (2020). Available online at: https://www.singstat.gov.sg/-/media/files/publications/cop2020/sr1/infographics.pdf (accessed October 20, 2021).

[44] Trubisky P Ting-Toomey S Lin SL. The influence of individualism-collectivism and self-monitoring on conflict styles. Int J Intercult. (1991) 15:65–84. 10.1016/0147-1767(91)90074-Q

[45] Tabachnick BG Fidell LS. Using Multivariate Statistics. 4th Edn. Needham Heights, MA: Allyn and Bacon (2001).

[46] Carr JZ Schmidt AM Ford JK DeShon RP. Climate perceptions matter: A meta-analytic path analysis relating molar climate, cognitive and affective states, and individual level work outcomes. J Appl Psychol. (2003) 88:605–19. 10.1037/0021-9010.88.4.605 12940402

[47] Steinmetz H. Analyzing observed composite differences across groups. Methodology. (2013) 9:1–12. 10.1027/1614-2241/a000049

[48] Parker CP Baltes BB Young SA Huff JW Altmann RA LaCost HA. Relationships between psychological climate perceptions and work outcomes: a meta-analytic review. J Organ Behav. (2003) 24:389–416. 10.1002/job.198

[49] Verjans-Janssen SR van de Kolk I Van Kann DH Kremers SP Gerards SM. Effectiveness of school-based physical activity and nutrition interventions with direct parental involvement on children's BMI and energy balance-related behaviors – A systematic review. PLoS ONE. (2018) 13:e0204560. 10.1371/journal.pone.0204560 30261057 PMC6160096

[50] Wäsche H Niermann C Bezold J Woll A. Family health climate: a qualitative exploration of everyday family life and health. BMC Public Health. (2021) 21:1261. 10.1186/s12889-021-11297-4 34187447 PMC8240432

[51] Rothbaum F Rosen K Uchida N Ujiie T. Family systems theory, attachment theory, and culture. Fam Process. (2002) 41:328–50. 10.1111/j.1545-5300.2002.41305.x 12395563

[52] Ho CL Mahirah D Ho CZH Thumboo J. The role of the family in health promotion: A scoping review of models, and mechanisms. Health Promotion Int [In print]. (2022). 10.1093/heapro/daac119 PMC9673498 36398941

[53] Ng RY Wong YS Yeo JY Koh CL Wilson C Gan SK. The associations between dietary practices and dietary quality, biological health indicators, perceived stress, religiosity, culture, and gender in multicultural Singapore. J Ethnic Foods. (2018) 5:220–7. 10.1016/j.jef.2018.07.003

[54] Yoon M Lai MHC. Testing factorial invariance with unbalanced samples. Struct Equ Model. (2018) 25:201–13. 10.1080/10705511.2017.1387859

