# Supplementary material for: The validation of culturally appropriate scales to assess the family health climate in a multi-ethnic Asian population
Source: Front Public Health. 2022 Oct 6;10:988525. doi: 10.3389/fpubh.2022.988525 (PMC9584639; doi:10.3389/fpubh.2022.988525)
Supplement: Supplementary File 1 — FHC-PA and NU (Sg) Chinese and Malay Final Versions. [file Data_Sheet_1.docx]

Supplementary Material

**Supplementary File 1. Final versions of the FHC (Sg) scales in Chinese and Malay**

**Chinese version**

**家庭健康意识调查问卷**

每行请只勾选一个框。

| FHC-PA (Sg)  在我们家... | 非常不同意 | 不太同意 | 还算同意 | 非常同意 |
| --- | --- | --- | --- | --- |
| Q1. ...我们注重让身体每天保持活跃（如散步、运动、参加体育活动)。 |  |  |  |  |
| Q2. ...经常保持身体活跃是很正常的。 |  |  |  |  |
| Q3. ...经常做运动对我们来说是正常的。 |  |  |  |  |
| Q4. ...在我们空闲的时候保持身体活跃是很正常的。 |  |  |  |  |
| Q5. ...我们都同意，身体运动是日常生活的一部分。 |  |  |  |  |
| Q6. ...我们互相鼓励支持保持身体活跃。 |  |  |  |  |
| Q7. …我们喜欢一起进行身体运动。 |  |  |  |  |
| Q8. ...我们喜欢一起运动。 |  |  |  |  |
| Q9. ...我们一起做身体活动时都很开心。 |  |  |  |  |
| Q10. ...我们觉得能一起做身体活动是很愉快的事。 |  |  |  |  |
| Q11. ...我们喜欢花时间在一起运动（如骑脚车、打球、划船）。 |  |  |  |  |
| Q12. ...我们通常会同意一起做的身体活动。 |  |  |  |  |
| Q13. …我们观看关于健身、身体活动或运动的视频（如Youtube、Netflix或电视节目）。 |  |  |  |  |
| Q14. ...我们会主动寻找身体活动和运动的最新资讯，随时了解最新消息。 |  |  |  |  |
| Q15. ...我们会收集健身、身体活动和运动方面的资讯（比如下载或者记下网上资料，剪下报刊文章）。 |  |  |  |  |
| Q16. ...我们会阅读报章杂志或网上有关健身、身体活动和运动方面的文章。 |  |  |  |  |
| FHC-NU (Sg)  在我们家... | 非常不同意 | 不太同意 | 还算同意 | 非常同意 |
| Q1. ...健康饮食对我们很重要。 |  |  |  |  |
| Q2. ...我们都很重视健康饮食。 |  |  |  |  |
| Q3. ...我们经常都会吃得健康。 |  |  |  |  |
| Q4. ...选择健康的食物对我们来说是正常的。 |  |  |  |  |
| Q5. ...我们对报刊或网上有关健康营养的文章感兴趣。 |  |  |  |  |
| Q6. ...我们会互相提醒，注意健康饮食。 |  |  |  |  |
| Q7. …我们会聊哪些食物是健康食物。 |  |  |  |  |
| Q8. ...我们互相鼓励和支持，避免吃不健康的食物和饮料。 |  |  |  |  |
| Q9. ...我们会聊到如何吃得健康。 |  |  |  |  |
| Q10. …我们珍惜一起用餐的时间。 |  |  |  |  |
| Q11. …大家都享受一起用餐。 |  |  |  |  |
| Q12. …一起用餐是我们日常家庭生活的一部分。 |  |  |  |  |
| Q13. …同桌用餐时，我们吃得最开心。 |  |  |  |  |
| Q14. …我们会尽可能经常一起用餐。 |  |  |  |  |
| Q15. ...我们对饮食和营养的意见一致（如食物的种类和分量，用餐时间等）。 |  |  |  |  |
| Q16. ...我们对餐饮的选择方面，一般都会意见相同。 |  |  |  |  |

**Malay version**

**SOAL SELIDIK IKLIM KESIHATAN KELUARGA**

Sila tandakan  SATU sahaja dalam setiap baris.

| FHC-PA (Sg)  Kami Sekeluarga… | Sangat Tidak Bersetuju | Agak Tidak Bersetuju | Agak Bersetuju | Sangat Bersetuju |
| --- | --- | --- | --- | --- |
| Q1. …kami memastikan yang kami aktif secara fizikal sepanjang semasa menjalani kehidupan seharian (contohnya berjalan-jalan, berkebun, bersenam, bersukan). |  |  |  |  |
| Q2. …adalah normal untuk aktif secara fizikal secara berkala. |  |  |  |  |
| Q3. …adalah normal untuk kami bersenam secara berkala. |  |  |  |  |
| Q4. …adalah normal untuk aktif secara fizikal pada masa lapang kami. |  |  |  |  |
| Q5. …kami bersetuju bahawa aktiviti fizikal adalah sebahagian daripada kehidupan seharian kami. |  |  |  |  |
| Q6. …kami menggalakkan dan menyokong antara satu sama lain untuk menjadi aktif secara fizikal. |  |  |  |  |
| Q7. …kami suka menghabiskan masa bersama melakukan aktiviti fizikal. |  |  |  |  |
| Q8. …kami seronok bersenam bersama. |  |  |  |  |
| Q9. … kami seronok melakukan aktiviti fizikal bersama. |  |  |  |  |
| Q10. …kami berasa sangat senang bersama melakukan aktiviti fizikal. |  |  |  |  |
| Q11. …kami suka menghabiskan masa bersama dalam sukan (contohnya berbasikal, permainan bola, berkanu). |  |  |  |  |
| Q12. …kami biasanya bersetuju dengan aktiviti fizikal untuk dilakukan bersama. |  |  |  |  |
| Q13. …kami menonton video (contohnya di Youtube, Netflix atau TV) tentang kecergasan, aktiviti fizikal atau senaman. |  |  |  |  |
| Q14 …kami secara aktif mencari maklumat terkini tentang aktiviti fizikal dan senaman untuk terus mengikuti perkembangan terkini. |  |  |  |  |
| Q15. …kami mengumpulkan maklumat (contohnya memuat turun/menanda maklumat dalam talian, memotong artikel cetak) tentang kecergasan, aktiviti fizikal dan senaman. |  |  |  |  |
| Q16. …kami membaca artikel (bercetak atau dalam talian) tentang kecergasan, aktiviti fizikal dan senaman. |  |  |  |  |
| FHC-NU (Sg)  Kami Sekeluarga… | Sangat Tidak Bersetuju | Agak Tidak Bersetuju | Agak Bersetuju | Sangat Bersetuju |
| Q1. …diet yang sihat adalah penting bagi kami. |  |  |  |  |
| Q2. …kami memberi perhatian untuk makan secara sihat. |  |  |  |  |
| Q3. …kami kerap makan secara sihat. |  |  |  |  |
| Q4. …adalah normal bagi kami untuk memilih makanan yang sihat. |  |  |  |  |
| Q5. …kami berminat dengan artikel (cetak atau dalam talian) tentang pemakanan yang sihat. |  |  |  |  |
| Q6. …kami saling mengingatkan antara satu sama lain untuk memberi perhatian kepada diet yang sihat. |  |  |  |  |
| Q7. …kami berbincang tentang makanan mana yang sihat. |  |  |  |  |
| Q8. …kami menggalakkan dan menyokong antara satu sama lain untuk menahan diri daripada makan/minum benda yang tidak sihat. |  |  |  |  |
| Q9. …kami berbincang tentang cara untuk makan secara sihat. |  |  |  |  |
| Q10. …kami menghargai menghabiskan masa bersama semasa makan. |  |  |  |  |
| Q11. …semua orang menikmati makan bersama. |  |  |  |  |
| Q12. …makan bersama adalah sebahagian daripada kehidupan keluarga seharian kami. |  |  |  |  |
| Q13. …kami paling menikmati makanan ketika duduk di meja yang sama. |  |  |  |  |
| Q14. …kami cuba makan bersama sekerap mungkin. |  |  |  |  |
| Q15. …kami bersetuju tentang diet dan pemakanan (contohnya jenis dan jumlah makanan, waktu makan). |  |  |  |  |
| Q16. …kami biasanya bersetuju dengan pilihan hidangan dan makanan. |  |  |  |  |

**Supplementary File 2. Family Health Climate - Physical Activity and Nutrition Scales (Singapore)**

**Version 1**

**Instructions:**

This questionnaire asks about your family’s attitudes and lifestyles towards physical activity and nutrition.

- Physical activity includes many ways of being active (e.g. taking walks, exercising, playing sports.
- Nutrition means eating a healthy and balanced diet (e.g. type and amount of food, meal timings).

Each statement below refers to the family members living with you **in the same household**. Choose an answer based on whether you agree or disagree with the statement as a description of your family. Select a response that comes quickly to mind. Try not to dwell on each question.

| FHC-PA (Sg)  In our family… | Strongly Disagree | Somewhat Disagree | Somewhat Agree | Strongly Agree |
| --- | --- | --- | --- | --- |
| … we make it a point of being physically active during our daily life (e.g. taking walks, gardening, exercising, playing sports). |  |  |  |  |
| … it is normal to be physically active on a regular basis. |  |  |  |  |
| … it is normal that we exercise on a regular basis. |  |  |  |  |
| …it is normal to be physically active in our free time. |  |  |  |  |
| … we agree that physical activities are part of our daily life. |  |  |  |  |
| … we like spending time together doing physical activities. |  |  |  |  |
| … we enjoy exercising together. |  |  |  |  |
| … we have fun doing physical activities together. |  |  |  |  |
| … we find it very pleasant to be together doing physical activities. |  |  |  |  |
| … we like spending time together in sports (e.g. cycling, ball games, canoeing). |  |  |  |  |
| … we watch videos (e.g. on Youtube, Netflix, or TV) on fitness, physical activities, or exercise. |  |  |  |  |
| … we actively look for the latest information on physical activity and exercise to stay up to date. |  |  |  |  |
| … we collect information (e.g. download/bookmark online information, cut out print articles) on fitness, physical activity and exercise. |  |  |  |  |
| … we read articles (printed or online) on fitness, physical activity and exercise. |  |  |  |  |
| … we encourage and support each other to be physically active. |  |  |  |  |
| … we usually agree on physical activities to do together. |  |  |  |  |
| FHC-NU (Sg)  In our family… | Strongly Disagree | Somewhat Disagree | Somewhat Agree | Strongly Agree |
| … a healthy diet is important to us. |  |  |  |  |
| … we pay attention to eating healthily. |  |  |  |  |
| … we regularly eat healthily. |  |  |  |  |
| … it is normal for us to choose healthy foods. |  |  |  |  |
| … we are interested in articles (print or online) on healthy nutrition. |  |  |  |  |
| … we remind each other to pay attention to a healthy diet. |  |  |  |  |
| … we talk about which foods are healthy. |  |  |  |  |
| … we encourage and support each other to refrain from eating/drinking unhealthy things. |  |  |  |  |
| … we talk about how to eat healthily. |  |  |  |  |
| … we value spending time together during meals. |  |  |  |  |
| … everybody enjoys having meals together. |  |  |  |  |
| … eating together is a part of our daily family life. |  |  |  |  |
| … we enjoy meals most when we sit at the same table. |  |  |  |  |
| … we try to eat together as often as possible. |  |  |  |  |
| … we rarely argue about food- or diet-related matters. |  |  |  |  |
| … we agree on diet and nutrition (e.g. type and amount of food, meal timings). |  |  |  |  |
| … we usually agree on meals and food choices. |  |  |  |  |

**Supplementary File 3. Short versions of the FHC-PA and -NU (Sg) scales**

We developed short versions of the scales to enable fast and simple assessments of the family health climate in situations when using the full versions might not be practical.

# Methods & Results

We first used the findings from Part B of the study to consider which items would be suitable to exclude. This included reviewing items with lower factor loadings within each construct and items that had similar or overlapping meanings. Upon consensus within the study team, 5 items were excluded from the FHC-PA (Sg) and FHC-NU (Sg) scales each, resulting in 11 items for the short versions.

The items that are included in the short versions can be found in Tables S1 and S2 below.

Confirmatory factor analysis was used to verify the factor structures and Cronbach’s alpha was used to determine the internal consistency reliability of the short versions:

The fit indices of the FHC-PA (Sg) short version scale (χ^2^=125.60, *df*=41, *p<.001*, χ^2^/*df*=3.06; SRMR=0.044; RMSEA=0.072; CFI=0.976; TLI=0.968) indicated an acceptable fit for the model. Factor loadings, item-total correlations and inter-factor correlations are shown in Tables S1 and S3. Cronbach’s alpha for the 11-item scale was 0.93 and internal consistency of the factors was very good, similar to the full version scale.

The fit indices of the FHC-NU (Sg) short version scale (χ^2^=67.41, *df*=38, *p*<.001, *χ^2^/df*=1.77; SRMR=0.029; RMSEA=0.044; CFI=0.989; TLI=0.985) indicated a good fit for the model. Factor loadings, item-total correlations and inter-factor correlations are shown in Tables S2 and S3. Cronbach’s alpha for the 11-item scale was 0.90 and internal consistency of the factors was excellent, similar to the full version scale.

We also refined the wordings of three items: Item 3 from short version FHC-PA (Sg) and Items 3 and 9 from short version FHC-NU (Sg). We tested the face validity of these items on 20 participants who were Singapore residents, aged 21 and above, through a 5-minute survey. The participants were recruited via convenience sampling through an online platform. They remained anonymous except for age, race and type of role within the household. All of them completed the short 5-minute survey, with a mean age of 30.3 years (*SD* = 6.85, range 23 to 54 years old). 85% were Chinese, 10% were Malay and 5% were Indian. In the survey, the participants were presented with the original phrasings alongside the rephrased items. For each comparison, they were asked if the old and new phrases were similar in meanings. If the meanings were perceived as different, participants were asked to explain their thoughts. They were also asked to express the items in their own words based on what they felt each item meant. 75% of the participants felt the phrasings did not differ in meaning. For the remainder, the perceived differences were minor, e.g. a few felt the inclusion of the words “for us” in the new phrasing for Item 3 of the FHC-PA (Sg) felt more inclusive of the family, and that the new phrase presented for Item 3 of the FHC-NU (Sg) emphasized the regular frequency compared to the old phrase.

# Conclusion

Future studies may utilise the FHC (Sg) short version scales as an alternative to the full version if time is limited. Overall the results of the confirmatory factor analyses and tests of internal consistency indicated that the short version scales were satisfactory and comparable to the full version.
